# Altering coenzyme specificity of *Pichia stipitis *xylose reductase by the semi-rational approach CASTing

**DOI:** 10.1186/1475-2859-6-36

**Published:** 2007-11-21

**Authors:** Ling Liang, Jingqing Zhang, Zhanglin Lin

**Affiliations:** 1 Department of Chemical Engineering, Tsinghua University, 1 Tsinghua Garden Road, Beijing 100084, China; 2 Department of Chemical Engineering, Massachusetts Institute of Technology, Cambridge, MA 02139, USA; 3 College of Life Sciences and Pharmaceutical Engineering, Nanjing University of Technology, 5 New Mofan Road, Nanjing, Jiangsu 210009, China

## Abstract

**Background:**

The NAD(P)H-dependent *Pichia stipitis *xylose reductase (PsXR) is one of the key enzymes for xylose fermentation, and has been cloned into the commonly used ethanol-producing yeast *Saccharomyces cerevisiae*. In order to eliminate the redox imbalance resulting from the preference of this enzyme toward NADPH, efforts have been made to alter the coenzyme specificity of PsXR by site-directed mutagenesis, with limited success. Given the industrial importance of PsXR, it is of interest to investigate further ways to create mutants of PsXR that prefers NADH rather than NADPH, by the alternative directed evolution approach.

**Results:**

Based on a homology model of PsXR, six residues were predicted to interact with the adenine ribose of NAD(P)H in PsXR and altered using a semi-rational mutagenesis approach (CASTing). Three rounds of saturation mutagenesis were carried to randomize these residues, and a microplate-based assay was applied in the screening. A best mutant 2-2C12, which carried four mutations K270S, N272P, S271G and R276F, was obtained. The mutant showed a preference toward NADH over NADPH by a factor of about 13-fold, or an improvement of about 42-fold, as measured by the ratio of the specificity constant *k*_cat_/*K*_*m*_^coenzyme^. Compared with the wild-type, the *k*_cat_^NADH ^for the best mutant was only slightly lower, while the *k*_cat_^NADPH ^decreased by a factor of about 10. Furthermore, the specific activity of 2-2C12 in the presence of NADH was 20.6 U·mg^-1^, which is highest among PsXR mutants reported.

**Conclusion:**

A seemingly simplistic and yet very effective mutagenesis approach, CASTing, was applied successfully to alter the NAD(P)H preference for *Pichia stipitis *xylose reductase, an important enzyme for xylose-fermenting yeast. The observed change in the NAD(P)H preference for this enzyme seems to have resulted from the altered active site that is more unfavorable for NADPH than NADH in terms of both *K*_*m *_and *k*_cat_. There are potentials for application of our PsXR in constructing a more balanced XR-XDH pathway in recombinant xylose-fermenting *S. cerevisiae *strains.

## Background

D-xylose is the second most abundant renewable sugar in nature, and its fermentation to ethanol is considered to have great economical potential. Unfortunately, *Saccharomyces cerevisiae*, which has been optimized for ethanol production, cannot utilize xylose efficiently, while D-xylulose, an isomerization product of D-xylose, can be assimilated. A major strategy for constructing xylose-fermenting *S. cerevisiae *is to introduce genes involved in xylose metabolism from other organisms. Xylose reductase (EC 1.1.1.21) and xylitol dehydrogenase (EC 1.1.1.9) from the xylose-fermenting yeast *Pichia stipitis*, have been cloned into *S. cerevisiae *to allow for xylose fermentation to ethanol [1]. In this case, xylose is converted into xylulose by the sequential actions of two oxidoreductases. First, PsXR (*Pichia stipitis *xylose reductase) catalyses the reduction of xylose into xylitol with NAD(P)H as co-substrate. Xylitol is then oxidized by PsXDH (*Pichia stipitis *xylitol dehydrogenase) which uses NAD^+ ^exclusively as co-substrate to yield xylulose. The different coenzyme specificity of the two enzymes PsXR and PsXDH, however, creates an intracellular redox imbalance, which results in low ethanol yields and considerable xylitol by-product formation. Efforts have been made to alter the coenzyme specificity of PsXR or PsXDH by site-directed mutagenesis [2-5]. The approach has worked well for PsXDH [4], while for PsXR the results were less successful, as the specific activity of the various mutants reported was significantly reduced [2,3]. Interestingly, however, Jeppsson et al expressed one of these PsXR mutants (K270M) in recombinant *S. cerevisiae *and observed increased ethanol levels accompanied by decreased xylitol yields [5]. Given the industrial importance of PsXR, it is of interest to further investigate ways to create mutants of PsXR that prefers NADH rather than NADPH by the alternative directed evolution approach.

XR belongs to the aldo-keto reductase (AKRs) superfamily, among which the enzymes have a high degree of amino acid sequence similarity [6]. Many crystallographic analyses of AKRs have revealed that they share a common (α/β)_8 _barrel fold, and with a highly conserved coenzyme binding pocket at the C-terminus. The nicotinamide ring of NAD(P)H is located at the core of the barrel, with the pyrophosphate bridge straddling the lip. Because NADH only differs from NADPH in the phosphate group esterified at the 2'-position of the adenosine ribose, a limited number of amino acid residues interacting with this part of the co-enzyme are the first candidates for engineering [7,8].

Although little structural knowledge of the PsXR is known, it has about 76% homology with the XR from *Candida tenuis *(CtXR), whose crystal structure is the only one reported among the yeast XRs and should provide clues for manipulating PsXR. In CtXR, the differences between the NAD^+^- and NADP^+^-bound forms of CtXR are located primarily in two regions. The first and largest of the conformational difference is in part of the loop in the eighth α/β motif. The second involves a smaller, but significant shift in a short helical region that appears at the end of β7 [9]. Sequence alignment results of PsXR and CtXR show that the amino acid residues interacting with the coenzymes are conserved, especially for the invariant IPKS (Ile-Pro-Lys-Ser) motif which is conserved in almost all yeast XRs [7]. Thus it is possible to predict the important residues that participate in the interactions of the coenzymes within PsXR based on the information from CtXR [8].

In this study, we altered the coenzyme specificity of PsXR by applying an iterative site-saturation mutagenesis method, CASTing (combinatorial active site saturation) to the predicted NAD(P)H binding region of PsXR. CASTing, first described by Reetz and co-workers [10], has been successfully used in engineering of a number of proteins [10-12]. The method is based on analyse of the three-dimensional structure of a target protein, and in each round of mutagenesis, two or three residues, whose side chains reside next to the binding pocket, are identified and randomized simultaneously to create relatively small libraries of mutants [13]. The best mutant thus obtained is used as the template for a further cycle of CASTing, until all the selected sites are randomized. In our study, six residues in PsXR were targeted, and three cycles of site-saturation mutagenesis were carried out.

## Results

### Cloning and expression of XR and NT-XR

**Figure 1:**
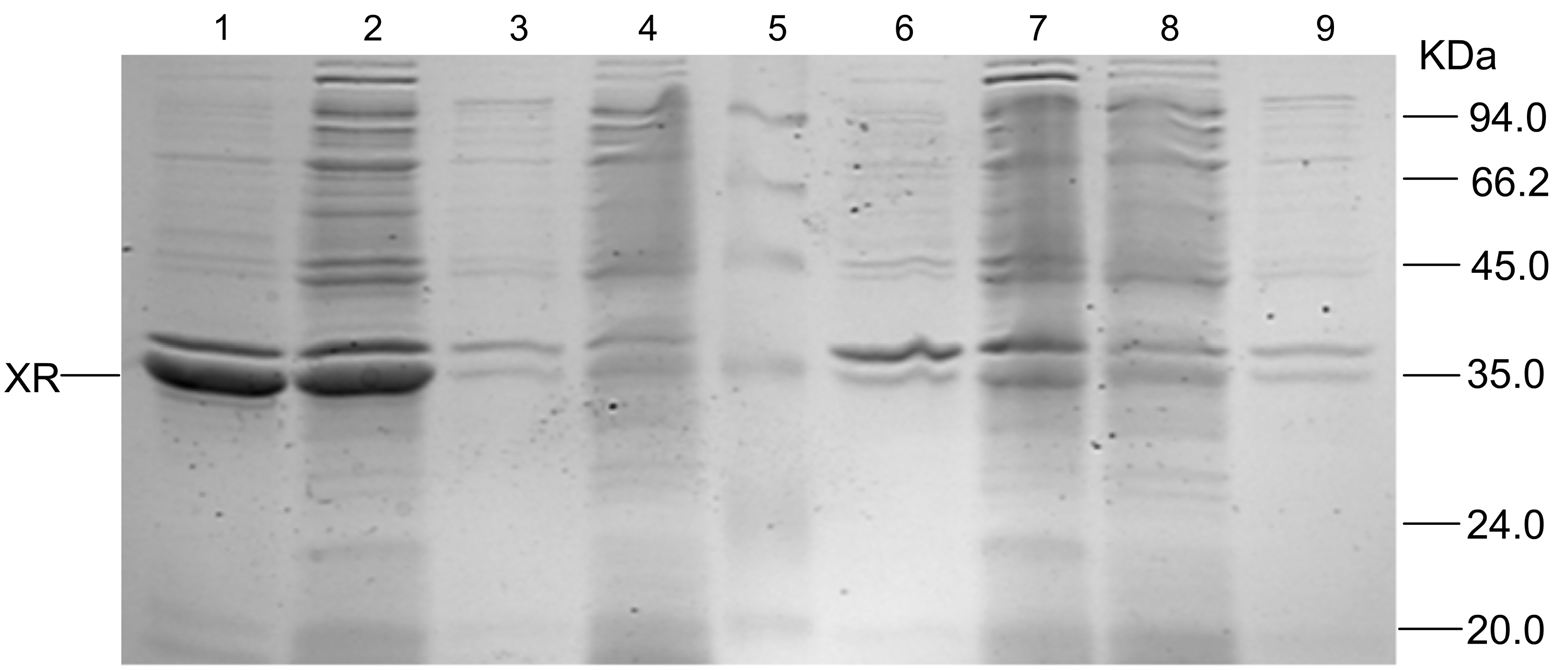
**SDS-PAGE analysis of crude extracts of XR, NT-XR and CT-XR**. Expression was carried out in *E. coli *cells in LB medium containing kanamycin (50 μg ml^-1^) and IPTG (1 mM). Crude extracts of different variants were adjusted to the same OD_600 _concentration (12.41 OD/ml) before they were prepared, and 15 μl was loaded in each lane. Lanes: 1, the insoluble fraction of NT-XR; 2, the soluble fraction of NT-XR; 3, the insoluble fraction of pET30a; 4, the soluble fraction of pET30a; 5, marker; 6, the insoluble fraction of XR; 7, the soluble fraction of XR; 8, the soluble fraction of CT-XR; 9, the insoluble fraction of CT-XR.

The PsXR encoding gene *XYL1 *was cloned by PCR from pMA91-*XYL1 *plasmid (X. Bao, unpublished data), ligated into the MCS of pET30a to yield pET30a-XR, and verified by sequencing. Using pET30a-XR as template, a His_6 _tag was introduced onto the N-terminus or C-terminus of PsXR, which yielded pET30a-NT-XR and pET30a-CT-XR, respectively. The expression level of NT-XR was about 5-fold higher than that of XR, while CT-XR showed little expression (Figure 1). In retrospect, this is consistent with the common structure of AKR members. The loops at the C-terminus are important for coenzyme binding [6], so the introduction of a His_6 _tag at the C-terminus of PsXR may affect the folding of the protein. pET30a-NT-XR was thus used as the starting point for selected site-saturation mutagenesis.

### The predicted NAD(P)H binding site of PsXR

**Figure 2:**
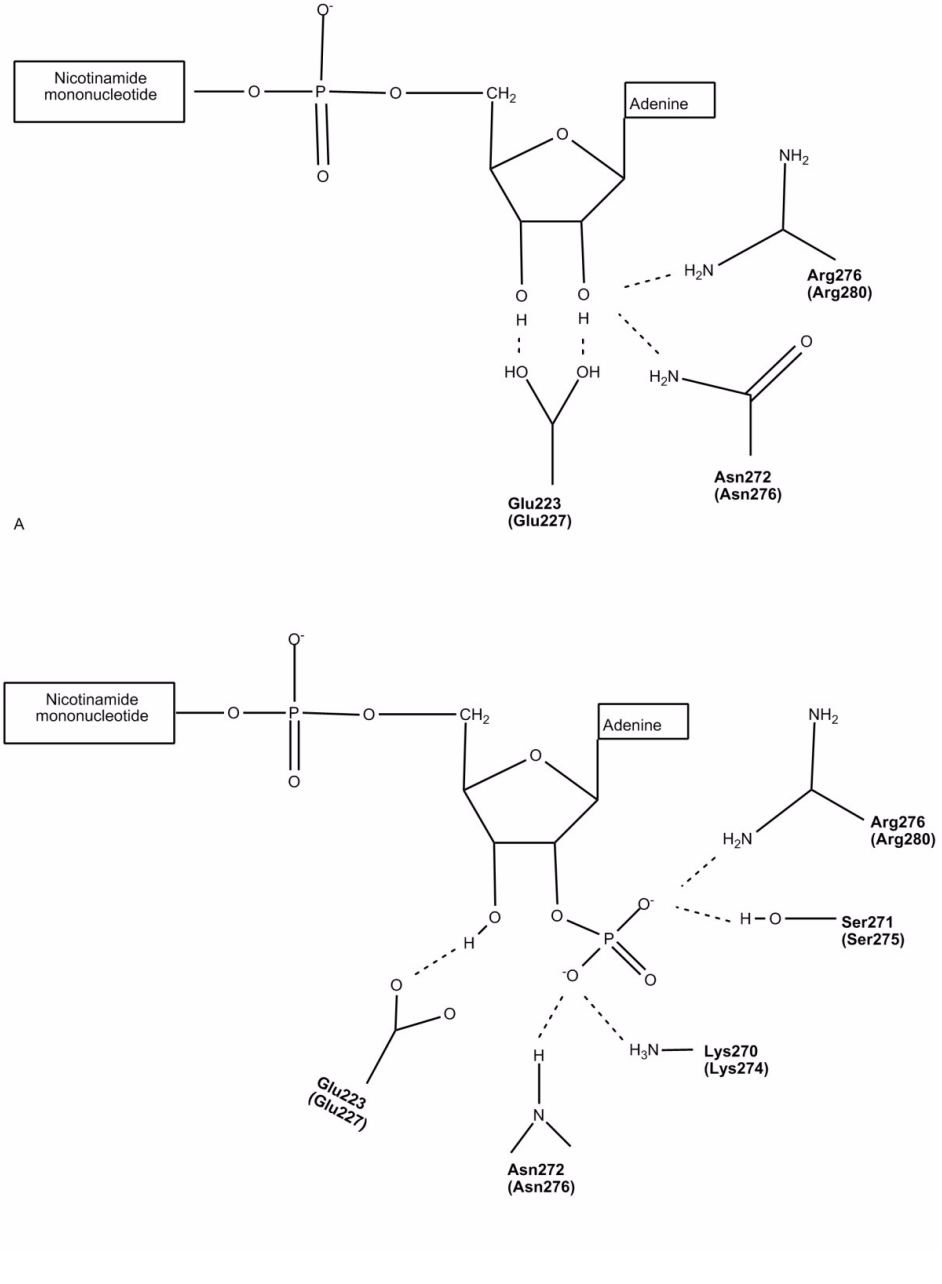
**Schematic diagrams showing the predicted interactions of the coenzymes with wild-type PsXR**. A) with NAD^+^; B) with NADP^+^. The predition is based on the coenzyme binding sites in CtXR [8]. And the residues predicted to be involved in coenzyme binding in PsXR are exactly the same as the corresponding CtXR residues (indicated in brackets). The corresponding CtXR residues are indicated in brackets.

As mentioned above, PsXR and CtXR share a high degree of homology at the amino acid level. Interactions between CtXR and coenzymes have been studied thoughtfully, and amino acid residues that participate in the coenzyme binding of CtXR have been mapped out [14]. These residues are conserved in PsXR. The amino acid sequence of PsXR was thus submitted to TASSER-Lite [15,16] to obtain a homology model for this protein based on CtXR, and residues that involve in coenzyme binding in PsXR were predicted and shown in Figure 2, with the corresponding CtXR residues indicated in brackets.

### Complete randomization of the predicted key sites involved in coenzyme binding

**Figure 3:**
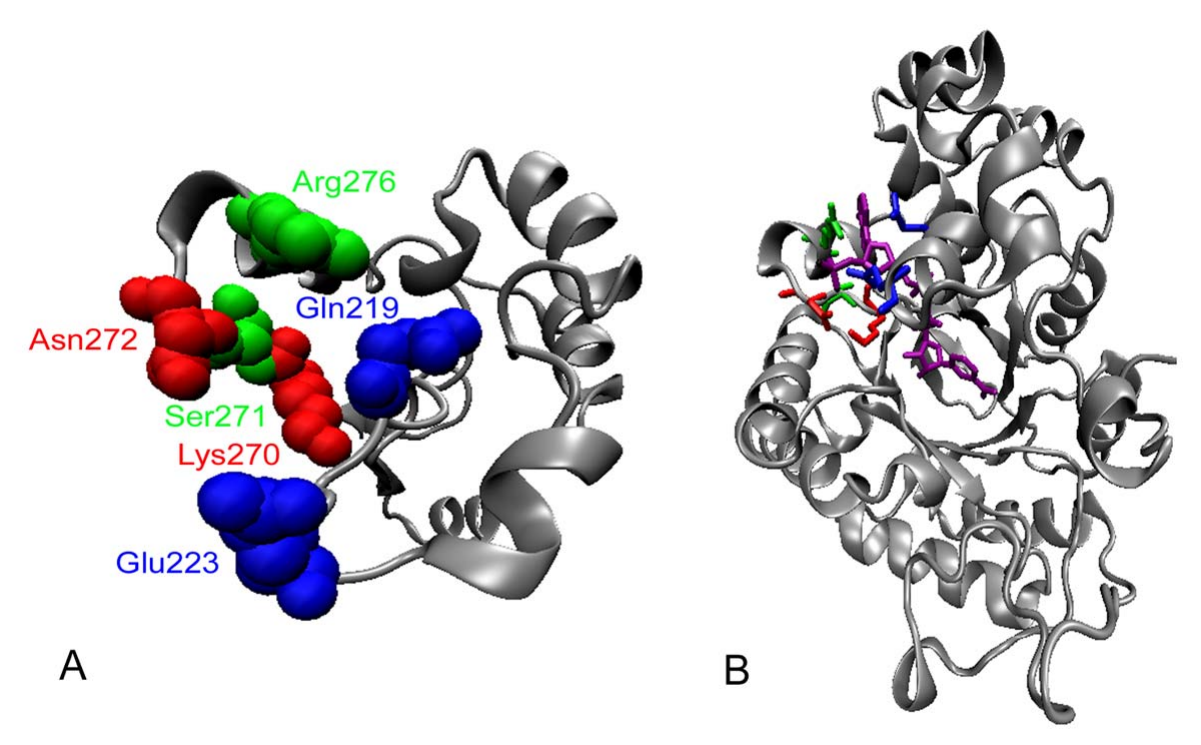
**Predicted coenzyme locations binding sites in PsXR**. A) Predicted locations in PsXR for residues that were chosen for CASTing. B) Predicted binding sites of wild-type NT-XR with NADPH. NADPH was in purple. The figures were prepared with VMD 1.8.4 [30], based on a structural model for PsXR obtained via homology modeling using the TASSER-Lite [15, 16, 31]. Red, green and blue colors indicate rounds 1, 2, 3 residues, respectively.

In applying the CASTing, six residues of PsXR were chosen as sites for randomization after careful examination of the predicted NAD(P)H binding pocket of PsXR, namely, Gln219, Glu223, Lys270, Ser271, Asn272, and Arg276 (Figure 3A). Gln219 was selected because its side chain is next to that of Glu223 in the three-dimensional structure, even though it has not been reported to be important in coenzyme binding. These six residues were grouped into three rounds of saturation mutagenesis in the order as follows: round 1 (Lys270, Asn272), round 2 (Ser271, Arg276), and round 3 (Gln219, Glu223). Round 1 and round 2 residues are located in the loop in the eighth α/β motif, and they all have interactions with the 2'-phosphate group of NADPH that is absent in NADH. Judging from previous site-directed mutageneses results on Lys270 in PsXR [3], and Lys274, Ser275, Asn276, Arg280, Lys274/Asn276, Lys274/Arg280 and Lys274/Asn276/Arg280 in CtXR [8,17] (the corresponding sites in PsXR are: Lys270, Ser271, Asn272, Arg276, Lys270/Asn272, Lys270/Arg276 and Lys270/Asn272/Arg276), it appeared that Lys270/Asn272 were most promising sites, thus these two residues were chosen for the first round of CASTing, followed by Ser271, Arg276 which also have direct contact with the 2'-phosphate group in NADPH. Round 3 residues are located at the end of β7, and have interactions with the 3'-hydroxy group of NAD(P)H. For CASTing experiments, the distance between the selected sites in each group is normally kept at less than 4 residues [13]. In this study, there are 4 amino acid residues between the two selected sites in round 2, but given that the side chains of these two residues are next to each other in the binding pocket (Figure 3), the choice of these residues fits the general principle of CASTing.

### Mutants with altered coenzyme specificity

In each cycle of saturation mutagenesis, NNK degeneracy was used (N stands for A, T, G or C, and K for T or G). Since only two amino acids were simultaneously randomized, the theoretical library size was 1,024, encoding 400 different protein variants. For each round, we screened about 1400 as described in the Methods section, and the coverage was over 75% as calculated by the traditional statistical method [18,19]. As indicated by Reetz et al, a lower coverage was often sufficient to find markedly improved mutants while reducing the screening effort [12,19,20]. For each round, about 10–20 variants whose initial activity ratio was significantly shifted toward NADH were re-screened by expressing the variants in the test tube, and the best mutant was used as the template for the next round. Table 1 showed the mutants obtained in the three cycles. The mutant 1-2F7 from the first round has two mutations K270S and N272P, while the mutant 2-2C12 from the second round has two more mutations, namely S271G and R276F. From the third cycle of mutagenesis, the best mutant 3-4A9 was found to have the same sequence as its template 2-2C12. So in our further study, the characterization of 3-4A9 was not determined.

**Table 1:** Construction of mutant PsXRs

Enzyme	Amino Acids^[a]^		PCR	
Wild-type NT-XR	GP**Q**SFV**E**LN	IP**KSN**TVP**R**L	Template	Primers^[b]^
1-2F7	·········	··S·P·····	NT-XR	5'-TCTTGGGACAGTMNNGGAMNNTGGAATGATGGCAATG-3'
				5'-CATCATTCCANNKTCCNNKACTGTCCCAAGATTGTTGGAAAAC-3'
2-2C12	·········	··SGP···F·	1-2F7	5'-TTTCCAACAAMNNTGGGACAGTAGGMNNCGATGGAATGA-3'
				5'-CATTCCATCGNNKCCTACTGTCCCANNKTTGTTGGAAAACA-3'
3-4A9	·········	··SGP···F·	2-2C12	5'-CCTTGGTTCAAMNNAACGAAAGAMNNAGGACCGAACGAAGAG-3'
				5'-GTTCGGTCCTNNKTCTTTCGTTNNKTTGAACCAAGGTAGAG-3'

[a] Dots indicate the same amino acid residues as PsXR. Bold letters show the randomized amino acid residues of PsXR in this study. [b] In the degenerate primers, underlines indicate mutated regions; M stands for A or C, K for T or G, and N for A, T, G or C. So in each round of saturation mutagenesis, the theoretical library size was (4*4*2)^2 = 1024.

According to the previous work on CtXR [8,17,21], the double mutant K270R/N272D showed the best NADH preference compared with the wild-type, however, during our CASTing experiments, we noticed that we missed this double mutant for PsXR in the first round of evolution. Thus we used site-directed mutagenesis to create this double mutant (denoted NT-K270R/N272D) and compared it to the two best mutants from first round and second round, respectively (mutants 1-2F7 and 2-2C12), using the microplate assay described in the experimental section. We found that this double mutant performed only marginally better than the mutant 1-2F7 but far worse than the mutant 2-2C12 in terms of activity ratio (NADH *vs *NADPH). It is thus not very surprisingly that we missed this double mutant NT-K270R/N272D in the first round of CASTing.

### Purification and characterization of recombinant PsXRs

Four PsXRs (wild-type NT-XR, mutants 1-2F7 and 2-2C12, and NT-K270R/N272D) with a His_6 _tag attached at their N-terminus were expressed in *E. coli *and purified with a Ni^2+^-chelating affinity column, and their kinetic characteristics were determined (Tables 2 and 3). As shown in Table 2, the introduction of a His_6 _tag at the N-terminus affected the specific activities with NADH and NADPH of wild-type PsXR by a factor of about 1.1 and 1.9, respectively; and as shown in Table 3, it also slightly affected the various *k*_cat_, *K*_*m*_, and *k*_cat_/*K*_*m *_values of the wild-type PsXR. In our work, for consistency, we used NT-XR as the benchmark for the comparison of kinetic parameters.

**Table 2:** Specific activity of wild-type and mutant PsXRs in this study.

	Specific activity [U·mg^-1^]		
Enzymes	NADH	NADPH	NADH-/NADPH-linked activity ratio
XR	16.7^[a]^	23.2^[a]^	0.72
Wild-type NT-XR	18.9 ± 1.3	44.1 ± 2.4	0.43
1-2F7	10.4 ± 0.4	10.8 ± 1.3	0.96
2-2C12	20.6 ± 2.3	0.82 ± 0.16	25.2
NT-K270R/N272D	19.3 ± 1.4	18.1 ± 1.7	1.07

[a] Data were quoted from C. V. K. Verduyn *et al *[32].

**Table 3:** Kinetic parameters of wild-type and mutant PsXRs for NADH and NADPH-dependent reactions

			NADH			NADPH		
Enzymes	*K*_*m *_for xylose (NADH) [mM]	*K*_*m *_for xylose (NADPH) [mM]	*K*_*m *_[mM]	*k*_cat _[Min^-1^]	*k*_cat_/*K*_*m *_[mM^-1^·min^-1^]	*K*_*m *_[mM]	*k*_cat _[Min^-1^]	*k*_cat_/*K*_*m *_[mM^-1^·min^-1^]
XR	42^[a]^	42^[a]^	0.021^[a]^	NA^[b]^	NA^[b]^	0.009^[a]^	NA^[b]^	167000^[c]^
Wild-type NT-XR	90 ± 7	82 ± 4	0.0106 ± 0.0006	923 ± 35	87630 ± 6730	0.0062 ± 0.0014	1650 ± 197	278900 ± 56300
1-2F7	314 ± 17	334 ± 56	0.512 ± 0.022	1110 ± 78	2180 ± 151	0.849 ± 0.045	2110 ± 236	2500 ± 300
2-2C12	291 ± 14	168 ± 22	0.147 ± 0.012	720 ± 57	4900 ± 315	0.427 ± 0.017	158 ± 9	370 ± 19
NT-K270R/N272D	128 ± 8	267 ± 31	0.058 ± 0.009	1075 ± 124	18800 ± 2400	0.142 ± 0.031	955 ± 203	6412 ± 1489

[a] Data were quoted from C. V. K. Verduyn *et al *[32]. [b] NA, not available. [c] Data were quoted from R. Woodyer *et al *[22].

The specific activities of wild-type NT-XR in the presence of NADH or NADPH were 18.9 and 44.1 U·mg^-1 ^(Table 2), respectively. For the mutant 2-2C12 obtained in the second cycle of CASTing, the specific activity in the presence of NADPH decreased by 53-fold compared to NT-XR, while the NADH-dependent specific activity was slightly better than that of NT-XR, and the ratio of NADH-/NADPH-dependent specific activity was changed from 0.43 for the wild-type to 25.2 for this mutant. On the other hand, the mutant 1-2F7 from the first and the double mutant NT-K270R/N272D showed a similar ratio of NADH-/NADPH-dependent specific activity of around 1.0, while the latter had higher NADH or NADPH-dependent specific activities than the former. As shown in Table 3, the mutations also affected the affinities for xylose. For the *K*_*m *_values of xylose in the presence of NADH, 1-2F7, 2-2C12 and K270R/N272D showed a 2.48-fold, 2.2-fold and 0.42-fold increase compared with wild-type NT-XR, respectively. While for the *K*_*m *_values of xylose in the presence of NADPH, 1-2F7, 2-2C12 and NT-K270R/N272D showed a 3.1-fold, 1.1-fold and 2.3-fold increase compared with wild-type NT-XR, respectively.

Kinetic constants for coenzymes of wild-type NT-XR and PsXR mutants are also shown in Table 3. Wild-type NT-XR showed a preference for NADPH over NADH with a *K*_*m *_value of 0.6-fold and a *k*_cat _value of 1.8-fold of those for NADH, respectively. As a consequence, NT-XR showed a 0.31 fold preference for NADH over NADPH, defined by the specificity constant ratio (*k*_cat_/*K*_*m*_^NADH^)/(*k*_cat_/*K*_*m*_^NADPH^). The mutant 1-2F7 exhibited a 136-fold increase in the *K*_*m *_value, and a 0.3-fold increase in the *k*_cat _value, for NADPH. Unexpectedly, the mutations also increase the *K*_*m *_and the *k*_cat _values by a factor of 47-fold and 0.2-fold, respectively, for NADH. As a result, for NADPH the specificity constant (*k*_cat_/*K*_*m*_^coenzyme^) of 1-2F7 was 0.009-fold of that of the wild-type, and for NADH this value is 0.025-fold. Thus the specificity constant ratio (*k*_cat_/*K*_*m*_^NADH^)/(*k*_cat_/*K*_*m*_^NADPH^) was shifted from 0.31 to 0.87. Compared with the wild-type NT-XR, the best mutant from the second round 2-2C12 showed a 67.8-fold increase in *K*_*m*_, accompanied by a 9.4-fold decrease in *k*_cat_, for NADPH. As for the NADH-dependent reaction, the mutations increased *K*_*m *_by 12.8-fold and decreased *k*_cat _only by a slight 0.2-fold. Thus 2-2C12 showed a 13.2-fold coenzyme preference for NADH over NADPH. This means that after two rounds of CASTing, the coenzyme preference for PsXR was significantly shifted by 42.6-fold toward NADH, again defined by the specificity constant ratio (*k*_cat_/*K*_*m*_^NADH^)/(*k*_cat_/*K*_*m*_^NADPH^).

On the other hand, compared with wild-type, the double mutant NT-K270R/N272D showed a 22-fold increase in the *K*_*m *_value, and a 0.7-fold decrease in the *k*_cat _value, for NADPH; and a 0.83-fold decrease in the *K*_*m *_value, and a 0.16-fold increase in the *k*_cat _value, for NADH. Thus it showed a 2.9-fold coenzyme preference for NADH over NADPH, which was higher than that of 1-2F7, but much lower than that of 2-2C12. This mutant also appeared in newly published work by Watanabe et al [21] (Table 4). While there is a discrepancy between our data set and the data set from Watanabe et al, these two data sets are in the same order of magnitude. We surmise that the difference in the N-terminal His_6 _tag of our mutant and that of Watanabe et al might affect the kinetic parameters of these two double mutants. It is also noteworthy that the Km values obtained in our work for wild type PsXR were close to the results (Table 4) previously reported (Table 4, column 4) [2,3].

**Table 4:** Comparison of the degree of alteration of coenzyme specificity for various mutants.

Enzyme	*K*_*m *_[mM]		*K*_*m*_^NADPH^/*K*_*m*_^NADH^	*k*_cat _[Min^-1^]		*k*_cat_^NADH^/*k*_cat_^NADPH^	(*k*_cat_/*K*_*m*_^NADH^)/(*k*_cat_/*K*_*m*_^NADPH^)
	NADH	NADPH		NADH	NADPH		
Wild-type NT-XR	0.0106	0.0062	0.58	923	1650	0.56	0.31
2-2C12	0.147	0.472	2.90	720	158	4.56	13.2
NT-K270R/N272D	0.058	0.142	2.45	1075	955	1.13	2.93

Wild-type^[a]^	0.021	0.009	0.43	NA^[d]^	NA^[d]^	NA^[d]^	NA^[d]^
Cys130Ser^[a]^	0.021	0.032	1.52	NA^[d]^	NA^[d]^	NA^[d]^	NA^[d]^
Wild-type^[b]^	0.028	0.026	0.93	NA^[d]^	NA^[d]^	NA^[d]^	NA^[d]^
Lys270Met^[b]^	0.031	0.131	4.30	NA^[d]^	NA^[d]^	NA^[d]^	NA^[d]^

Wild-type^[c]^	0.0305	0.0025	0.082	415	630	0.66	0.05
R276H^[c]^	0.017	0.0017	0.1	408	16	25.5	2.62
K270R/N272D^[c]^	0.138	2.81	20.4	706	1850	0.65	7.29

[a] Data were quoted from Y. Y. Zhang *et al *[2]. [b] Data were quoted from M. Kostrzynska *et al*, and the substrate used in the reaction mixture was DL-glyceraldehyde instead of xylose [3]. [c] Data were quoted from S. Watanabe *et al *[21]. [d] NA, not available.

## Discussion

To understand the reasons for the shift in the coenzyme specificity of PsXR, we analyzed the four mutations in 2-2C12, K270S, S271G, N272P and R276F. These mutations share some common features. The general trend of mutagenesis is from residues with long side chains to those with shorter side chains, and from hydrophilic ones to less hydrophilic ones, thus part of the coenzyme binding pocket involved in direct NAD(P)H contact becomes larger, which might have made the binding with NAD(P)H less favourable, and in consequence led to increases in the *K*_*m *_values for NAD(P)H. On the other hand, glycine and proline are known to frequently occur in turn regions of proteins, so mutations S271G and N272P might cause turns in the loop, which could led to a reduced coenzyme binding pocket that favours NADH. As a result of these mutations, mutant 2-2C12 shows a 12.8-fold increase for NADH and a 67.8-fold increase for NADPH in the *K*_*m *_value. What is more, the catalytic constant *k*_cat _for NADH is only slightly lower. But for NADPH, the *k*_cat _exhibits a 9.4-fold decrease. These data support the idea that the mutations were more harmful to NADPH binding and catalysis. The altered coenzyme specificity in PsXR by iterative saturation mutagenesis also supports the prediction that the loop in the eighth α/β repeat is a major determinant of NAD(P)H recognition.

In the third cycle of saturation mutagenesis, Gln219 and Glu223 at the end of β7, which have interactions with the 3'-hydroxy group of NAD(P)H, were randomized. However, no mutant with improved coenzyme specificity for NADH over NADPH was obtained. Perhaps it is because Glu223 plays a role in interactions of PsXR with both NADH and NADPH. In the case of NADH binding, Glu223 also makes close contact with the 2'-hydroxy group of the ribose group and acts as a hydrogen-bond acceptor. Alteration of this site might be more harmful to the binding of NADH. However, if one starts the iterative CASTing process by first focusing on these two sites, the outcome might be quite different, as the potential harmful effect could be compensated by subsequent mutations.

Although the *k*_cat _value of mutant 2-2C12 is a little lower than the wild-type NT-XR, this value is comparable among xylose reductases. For native XRs, the *k*_cat _for NADH is about 310 min^-1 ^in the case of *Neurospora crassa *XR, and 3100 min^-1 ^for the NADH-specific *Candida parapsilosis *XR [22]. And for the XR with coenzyme specificity successfully changed to favor NADH, the *k*_cat _is 720 min^-1 ^in the case of *Candida tenuis *XR mutant (K274R/N276D) [8], and is 706 min^-1 ^for the PsXR mutant K270R/N272D [21].

In the previous published work, Cys130 in [2] and Lys270 in [3] in PsXR were mutated, and *K*_*m *_values for coenzymes were measured. Judging from the *K*_*m*_^NADPH^/*K*_*m*_^NADH ^values (Table 4), both mutant C130S and K270M altered the ratio by a factor of 3.5 to 4.6, rather similar to 2-2C12 in our work. However, C130S lost about 70% of the specific activity as compared to wild-type [2], and for the mutant K270M, the specific activity decreased by 80–90% [3], while for 2-2C12 in our work, the specific activity with NADH (20.6 U·mg^-1^) was actually slightly higher than that of wild-type NT-XR.

Recently, Watanabe et al published results on a single mutant R276H and a double mutant K270R/N272D [21]. According to their measurement, the *K*_*m *_ratio (*K*_*m*_^NADPH^/*K*_*m*_^NADH^) was 0.1 for R276H, and 20.4 for K270R/N272D; the *k*_cat _ratio (*k*_cat_^NADH^/*k*_cat_^NADPH^) was 25.5 for R276H, and 0.65 for K270R/N272D; and lastly the specificity constant ratio ((*k*_cat_/*K*_*m*_^NADH^)/(*k*_cat_/*K*_*m*_^NADPH^)) was 2.62 for R276H, and 7.29 for K270R/N272D (Table 4). In addition, the specific activity ratio (in the presence of NADH *vs *NADPH) was 23.1 for R270H, and 3.9 forK270R/N272D [21]. In batch fermentation, it was found that R276H performed better than K270R/N272D in terms of ethanol yield and xylitol accumulation. In comparison, for 2-2C12 obtained in this work, the ratios of *K*_*m*_, *k*_cat_, *k*_cat_/*K*_*m*_, and specific activity (NADH *vs *NADPH) were 2.9, 4.56, 13.2, and 25.2, respectively (Tables 2 and 4), it would be interesting to see how this mutant would perform in batch fermentation, which should guide further engineering of this enzyme.

Along this line, it is noteworthy that *k*_cat_/*K*_*m*_^NADH ^decreased by a factor of about 17 fold for 2-2C12. However, as pointed by Watanabe et al, the intracellular activity of the enzyme are sensitive to changes in *k*_cat_, but much less so to changes in *K*_*m *_[21]. This observation implies that intracellular NAD(P)H concentrations are likely in the range of a few hundred μM or so, comparable to the *K*_*m *_values of the various PsXR mutants determined *in vitro*. Indeed, it has been shown that in *S. cerevisiae *cells the intracellular concentrations of NADH in microaerobic batch cultures are about 0.36 mM during the mid exponential growth phase and 0.15 mM during the stationary phase [23], although the information on intracellular NADH concentrations in *S. cerevisiae *under anaerobic conditions is unavailable. Therefore, in theory, the increased *K*_*m *_value in mutant 2-2C12 might not significantly affect the activity for the enzyme *in vivo*.

## Conclusion

The alteration of cofactor specificity in PsXR by iterative saturation mutagenesis supports the hypothesis that the loop in the eighth α/β repeat is a major determinant of NAD(P)H recognition. There are obvious potentials for application of our PsXR in constructing recombinant xylose-fermenting *S. cerevisiae *strains. Two common pathways for metabolism of xylose in *S. cerevisiae *have been adopted: the XR-XDH pathway, and the xylose isomerase (XI) pathway. Until now, only a few XIs could express at high activity levels in *S. cerevisiae *[24,25], so it is useful to exploit further the XR-XDH pathway. A highly NADH-preferred PsXR can help to eliminate the redox imbalances created during xylose assimilation using PsXR and PsXDH. In our further work, mutated PsXR will be cloned into recombinant xylose-fermenting *S. cerevisiae *together with PsXDH, to construct a new pathway to reduce the redox imbalance.

## Methods

### Materials

Restriction enzymes, DNA-modifying enzymes, and DNA polymerase were from New England Biolabs (Beverly, MA), except for Pfu DNA polymerase, which was purchased from Tiangen (Beijing, China). Lysozyme was from Sigma Chemical Co. (St. Louis, MO) and Dnase I was from Takara (Dalian, China). Oligonucleotides were either synthesized from Sangon (Shanghai, China) or Takara. Sequence analysis was either performed by Takara or by Sunbiotech Co., Ltd. (Beijing, China). The kits for DNA purification, gel recovery and plasmid mini-prep were either from Tiangen or QIAgen (Valencia, CA). *Escherichia coli *strain BL21(DE3) and pET30a(+) came from Novagen (Madison, WI). Isopropylthio-β-D-galactoside (IPTG) was obtained from Takara.

### Cloning of PsXR gene

Plasmid pMA91 harboring *XYL1 *gene was kindly provided by Prof. Xiaoming Bao, Shandong University, China. Based on the published sequence of the *P. stipitis XYL1 *gene [GenBank: X59465] [26], the following two primers were designed to span the full code region of this gene: XYL1^UP^, 5'-GCCTTCCCATATGCCTTCTATTAAGTTGAACTCT-3' and XYL1^DOWN^, 5'-GGAATCTCGAGTTAGACGAAGATAGGAATCTTGTC-3' (*Nde *I and *Xho *I sites are underlined, respectively). PCR reaction was carried out for 30 cycles using Pfu DNA polymerase in a 50 μl volume under the following conditions: 94°C for 1 min, 59°C for 1 min, and 72°C for 2 min. PCR product was purified, digested and inserted into the *Nde *I – *Xho *I sites in pET30a(+) to yield pET30a-XR. *E. coli *BL21 (DE3) cells were used throughout for cloning and PsXR expression.

To introduce an N-terminal or C-terminal His_6 _tag to *XYL1 *gene for protein purification, PCR were carried out using pET30a-XR as the template DNA and the following pairs of primers: NT-XYL1^UP^, 5'-GATAAGTCATATG*CACCACCACCACCACCAC***AGCAGTGCT**ATGCCTTCTATTAAGTTGA-3' and XYL1^DOWN ^(for the N-terminal His_6 _tag); XYL1^UP ^and CT-XYL1^DOWN^, 5'-AACATCTCGAGTTA*GTGGTGGTGGTGGTGGTG***GGAAGCAGA**GACGAAGATAGGAATCTT-3' (for the C-terminal His_6 _tag), *Nde *I and *Xho *I sites are underlined, His_6 _tags are in italic, and the linker sequences are in bold. For the C-terminal His_6 _tag, in order to avoid the extra residues after the His_6 _tag in the pET30a(+), primer overhangs were thus used to introduce the His_6 _tag. The amplified PCR fragments were introduced into the *Nde *I – *Xho *I sites in pET30a(+) to yield pET30a-NT-XR or pET30a-CT-XR, respectively.

### Site-saturation mutagenesis (CASTing) and K270R/N272D construction

The amino acid mutations were introduced sequentially by overlapping PCR. In the first round, two reactions, I and II, were performed using pET30a-NT-XR as template with the following primers, for reaction I using NT-XYL1^UP ^and SSM1st^1^, 5'-TCTTGGGACAGTMNNGGAMNNTGGAATGATGGCAATG-3'; and for reaction II using XYL1^DOWN ^and SSM1st^2^, 5'-CATCATTCCANNKTCCNNKACTGTCCCAAGATTGTTGGAAAAC-3' (mutated positions are underlined, M stands for A or C, K for T or G, and N for A, T, G or C). The amplified DNA fragments of reaction I and II were treated with *Dpn *I respectively, and then overlapped for 10 cycles under the following conditions: 94°C for 1 min, 69°C for 1 min, and 72°C for 2 min. The full length mutated gene was amplified with primers NT-XYL1^UP ^and XYL1^DOWN ^under 20 cycles of 94°C for 1 min, 50°C for 1 min, and 72°C for 2 min, and inserted into the *Nde*I-*Xho*I sites in pET30a(+) to yield pET30a-SSM1st. The second and third rounds of CASTing were carried out similarly using the following primers, SSM2nd^1^, 5'-TTTCCAACAAMNNTGGGACAGTAGGMNNCGATGGAATGA-3' and SSM2nd^2^, 5'-CATTCCATCGNNKCCTACTGTCCCANNKTTGTTGGAAAACA-3' (for the second round); SSM3rd^1^, 5'-CCTTGGTTCAAMNNAACGAAAGAMNNAGGACCGAACGAAGAG-3' and SSM3rd^2^, 5'-GTTCGGTCCTNNKTCTTTCGTTNNKTTGAACCAAGGTAGAG-3' (for the third round).

To generate the double mutant K270R/N272D, pET30a-NT-XR was used as the template. And overlapping PCR were carried out similarly using the following primers, RD^1^, 5'-CATCATTCCAAGATCCGATACTGTCCCAAGA-3' and RD^2^, 5'-TTGGGACAGTATCGGATCTTGGAATGATGGCAATGC-3' (mutated positions are underlined).

### Screening for the saturation mutagenesis library

Plasmids were transformed into *E. coli *BL21(DE3), and individual colonies were used to inoculate LB medium (200 μl) supplemented with kanamycin (50 μg ml^-1^) in 96-well microtiter plates. The plates were grown at 37°C overnight; 5 μl of each culture was used to inoculate a duplicate plate for expression. After 3 h of growth at 37°C, IPTG was added to a final concentration of 0.5 mM, and protein expression was carried out at 30°C for 6 h. Cells were harvested by centrifugation at 1,700 g for 20 min at 4°C, and the pellets were first stored at -70°C overnight, resuspended in 200 μl lysis buffer (50 mM potassium buffer, pH 6.0, 1 mg ml^-1 ^lysozyme and 2 U ml^-1 ^Dnase I), and then incubated at 37°C for 60 min. Cell lysates were centrifuged at 1,700 g for 20 min at 4°C and supernatants were used directly for XR assay as follows: 20 μl of supernatants was added into 180 μl of reaction mixture (50 mM potassium phosphate buffer, pH 6.0, containing 0.5 mM NAD(P)H and 0.2 M xylose) in a 96-well plate. Reactions were incubated at 30°C in the chamber of a SpectroMAX 190 Microtiter reader (Molecular Devices, CA), and absorbance at 340 nm was measured at 20 s intervals for 20 min using the NAD(P)H depletion assay [27,28]. Variants which had the highest ratios of activity with respect to NADH against that with respect to NADPH were chosen for re-screening in the test tube.

### Expression and purification of His_6_-tagged PsXRs

*E. coli *BL21(DE3) harbouring the expression plasmids for the His_6_-tagged wild-type enzyme (WT) and mutated PsXRs were grown at 37°C overnight in LB medium containing kanamycin (50 μg ml^-1^). The saturated overnight cultures were diluted 100-fold into fresh LB media containing kanamycin (50 μg ml^-1^) and grown at 37°C for about 2 h to reach an OD_600 _of 0.5~0.6. Protein expression was initiated with 0.5 mM IPTG, and continued for 6 h at 30°C. Cells were then harvested and resuspended in 30 ml of Buffer A (20 mM sodium phosphate, pH 7.4, containing 500 mM NaCl and 30 mM imidazole) and subjected to 99 pulses of sonication, 4 s each with a 3 s interval in an ice-water bath. After centrifugation, the supernatant was passed through a 0.22 μm low binding membrane (Dingguo, Beijing, China). All purification experiments were carried out using an ÄKTA purifier system using a Ni^2+^-charged 1 ml HiTrap Chelating HP column equilibrated with Buffer A. Enzymes were eluted with Buffer B (Buffer A plus 500 mM imidazole). Recombinant PsXRs (wild-type and mutants) were stored at -70°C in 20% glycerol until use.

### Kinetic characterization of PsXRs

All kinetic parameters were determined in potassium phosphate buffer (50 mM, pH 6.0). Enzymes were used at concentrations ranging from 20 to 1200 nM. When assaying activity with varied xylose, NAD(P)H was kept at 0.5 mM, and when assaying activity with varied NAD(P)H, xylose was kept at 0.5 M. Activity was determined by measuring the decrease in absorbance at 340 nm corresponding to the oxidization of NAD(P)H to NAD(P)^+ ^using a SpectroMAX 190 Microtiter reader. Enzyme units were defined as μmol of NAD(P)H reduced per minute. The kinetic parameters, *K*_*m *_and *k*_cat _values, were calculated by Lineweaver-Burk plot. Protein concentrations were determined using absorbance at 280 nm [29].

## Abbreviations used

CtXR : *Candida tenuis *xylose reductase;

PsXDH : *Pichia stipitis *xylitol dehydrogenase;

PsXR : *Pichia stipitis *xylose reductase;

XDH : Xylitol dehydrogenase;

XI : Xylose isomerase.

XR : Xylose reductase;     

## Competing interests

The author(s) declare that they have no competing interests.

## Authors' contributions

LL constructed the homology model, carried out the cloning, three rounds of saturation mutagenesis and kinetic characterization, and drafted the manuscript. JZ participated in kinetic characterization and helped with the manuscript preparation. ZL conceived the study, designed and supervised the experiments, and revised the manuscript.

## References

[B1] Kötter P, Ciriacy M (1993). Xylose fermentation by *Saccharomyces cerevisiae*. Appl Microbiol Biotechnol.

[B2] Zhang YY, Lee H (1997). Site-directed mutagenesis of the cysteine residues in the *Pichia stipitis* xylose reductase. FEMS Microbiol Lett.

[B3] Kostrzynska M, Sopher CR, Lee H (1998). Mutational analysis of the role of the conserved lysine-270 in the *Pichia stipitis* xylose reductase. FEMS Microbiol Lett.

[B4] Watanabe S, Kodaki T, Makino K (2005). Complete reversal of coenzyme specificity of xylitol dehydrogenase and increase of thermostability by the introduction of structural zinc. J Biol Chem.

[B5] Jeppsson M, Bengtsson O, Franke K, Lee H, Hahn-Hagerdal R, Gorwa-Grauslund MF (2006). The expression of a *Pichia stipitis* xylose reductase mutant with higher *K_m_* for NADPH increases ethanol production from xylose in recombinant *Saccharomyces cerevisiae*. Biotechnol Bioeng.

[B6] Jez JM, Bennett MJ, Schlegel BP, Lewis M, Penning TM (1997). Comparative anatomy of the aldo-keto reductase superfamily. Biochem J.

[B7] Lee H (1998). The structure and function of yeast xylose (aldose) reductases. Yeast.

[B8] Petschacher B, Leitgeb S, Kavanagh KL, Wilson DK, Nidetzky B (2005). The coenzyme specificity of *Candida tenuis* xylose reductase (AKR2B5) explored by site-directed mutagenesis and X-ray crystallography. Biochem J.

[B9] Kavanagh KL, Klimacek M, Nidetzky B, Wilson DK (2003). Structure of xylose reductase bound to NAD(+) and the basis for single and dual co-substrate specificity in family 2 aldo-keto reductases. Biochem J.

[B10] Reetz MT, Gates B, Knözinger H (2006). Directed evolution of enantioselective enzymes as catalysts for organic synthesis. Advances in Catalysis.

[B11] Reetz MT, D Carballeira J, Vogel A (2006). Iterative saturation mutagenesis on the basis of B factors as a strategy for increasing protein thermostability. Angew Chem, Int Ed.

[B12] Reetz MT, Wang LW, Bocola M (2006). Directed evolution of enantioselective enzymes: iterative cycles of CASTing for probing protein-sequence space. Angew Chem, Int Ed.

[B13] Reetz MT, Bocola M, Carballeira JD, Zha DX, Vogel A (2005). Expanding the range of substrate acceptance of enzymes: combinatorial active-site saturation test. Angew Chem, Int Ed.

[B14] Kavanagh KL, Klimacek M, Nidetzky B, Wilson DK (2002). The structure of apo and holo forms of xylose reductase, a dimeric aldo-keto reductase from *Candida tenuis*. Biochemistry.

[B15] Skolnick J, Kihara D, Zhang Y (2004). Development and large scale benchmark testing of the PROSPECTOR_3 threading algorithm. Proteins: Struct, Funct, Bioinf.

[B16] Zhang Y, Skolnick J (2004). Automated structure prediction of weakly homologous proteins on a genomic scale. Proc Natl Acad Sci U S A.

[B17] Petschacher B, Nidetzky B (2005). Engineering *Candida tenuis* xylose reductase for improved utilization of NADH: antagonistic effects of multiple side chain replacements and performance of site-directed mutants under simulated *in vivo* conditions. Appl Environ Microbiol.

[B18] Warren MS, Benkovic SJ (1997). Combinatorial manipulation of three key active site residues in glycinamide ribonucleotide transformylase. Protein Eng.

[B19] Reetz MT, Carballeira JD (2007). Iterative saturation mutagenesis (ISM) for rapid directed evolution of functional enzymes. Nat Protocols.

[B20] Reetz MT, Wilensek S, Zha DX, Jaeger KE (2001). Directed evolution of an enantioselective enzyme through combinatorial multiple-cassette mutagenesis. Angew Chem, Int Ed.

[B21] Watanabe S, Abu Saleh A, Pack SP, Annaluru N, Kodaki T, Makino K (2007). Ethanol production from xylose by recombinant *Saccharomyces cerevisiae* expressing protein-engineered NADH-preferring xylose reductase from *Pichia stipitis*. Microbiology.

[B22] Woodyer R, Simurdiak M, van der Donk WA, Zhao HM (2005). Heterologous expression, purification, and characterization of a highly active xylose reductase from *Neurospora crassa*. Appl Environ Microbiol.

[B23] Heux S, Cachon R, Dequin S (2006). Cofactor engineering in *Saccharomyces cerevisiae*: expression of a H_2_O-forming NADH oxidase and impact on redox metabolism. Metab Eng.

[B24] Walfridsson M, Anderlund M, Bao X, HahnHagerdal B (1997). Expression of different levels of enzymes from the *Pichia stipitis* XYL1 and XYL2 genes in *Saccharomyces cerevisiae* and its effects on product formation during xylose utilisation. Appl Microbiol Biotechnol.

[B25] Kuyper M, Harhangi HR, Stave AK, Winkler AA, Jetten MSM, de Laat W, den Ridder JJJ, Op den Camp HJM, van Dijken JP, Pronk JT (2003). High-level functional expression of a fungal xylose isomerase: the key to efficient ethanolic fermentation of xylose by *Saccharomyces cerevisiae*?. FEMS Yeast Res.

[B26] Amore R, Kotter P, Kuster C, Ciriacy M, Hollenberg CP (1991). Cloning and expression in *Saccharomyces cerevisiae* of the NAD(P)H-dependent xylose reductase-encoding gene (XYL1) from the xylose-assimilating yeast *Pichia stipitis*. Gene.

[B27] Glieder A, Meinhold P, Arnold FH, Georgiou G (2003). High-throughput screens based on NAD(P)H depletion. Directed Enzyme Evolution: Screening and Selection Methods.

[B28] O'Hare HM, Baerga-Ortiz A, Popovic B, Spencer JB, Leadlay PF (2006). High-throughput mutagenesis to evaluate models of stereochemical control in ketoreductase domains from the erythromycin polyketide synthase. Chem Biol.

[B29] Sapan CV, Lundblad RL, Price NC (1999). Colorimetric protein assay techniques. Biotechnol Appl Biochem.

[B30] Humphrey W, Dalke A, Schulten K (1996). VMD: visual molecular dynamics. J Mol Graphics.

[B31] TASSER-Lite: protein structure prediction and modeling tool (beta-release). http://cssb.biology.gatech.edu/skolnick/webservice/tasserlite/index.html.

[B32] Verduyn C, Van Kleef R, Frank J, Schreuder H, Van Dijken JP, Scheffers WA (1985). Properties of the NAD(P)H-dependent xylose reductase from the xylose-fermenting yeast *Pichia stipitis*. Biochem J.

